# Multivariate Analysis of ^18^F-DMFP PET Data to Assist the Diagnosis of Parkinsonism

**DOI:** 10.3389/fninf.2017.00023

**Published:** 2017-03-30

**Authors:** Fermín Segovia, Juan M. Górriz, Javier Ramírez, Francisco J. Martínez-Murcia, Johannes Levin, Madeleine Schuberth, Matthias Brendel, Axel Rominger, Kai Bötzel, Gaëtan Garraux, Christophe Phillips

**Affiliations:** ^1^Department of Signal Theory, Networking and Communications, University of GranadaGranada, Spain; ^2^Cyclotron Research Centre, University of LiègeLiège, Belgium; ^3^Department of Neurology, University of MunichMunich, Germany; ^4^Department of Nuclear Medicine, University of MunichMunich, Germany

**Keywords:** multivariate analysis, ^18^F-DMFP PET, Parkinson's disease, multiple kernel learning, support vector machine, multiple system atrophy, progressive supranuclear palsy

## Abstract

An early and differential diagnosis of parkinsonian syndromes still remains a challenge mainly due to the similarity of their symptoms during the onset of the disease. Recently, ^18^F-Desmethoxyfallypride (DMFP) has been suggested to increase the diagnostic precision as it is an effective radioligand that allows us to analyze post-synaptic dopamine *D*_2/3_ receptors. Nevertheless, the analysis of these data is still poorly covered and its use limited. In order to address this challenge, this paper shows a novel model to automatically distinguish idiopathic parkinsonism from non-idiopathic variants using DMFP data. The proposed method is based on a multiple kernel support vector machine and uses the linear version of this classifier to identify some regions of interest: the olfactory bulb, thalamus, and supplementary motor area. We evaluated the proposed model for both, the binary separation of idiopathic and non-idiopathic parkinsonism and the multigroup separation of parkinsonian variants. These systems achieved accuracy rates higher than 70%, outperforming DaTSCAN neuroimages for this purpose. In addition, a system that combined DaTSCAN and DMFP data was assessed.

## 1. Introduction

Parkinson's disease (PD) has a lifetime risk of about 2%, making it the second most common neurodegenerative disease after Alzheimer's disease. A main risk factor for PD is aging and current demographic trends predict a doubling in the number of cases by 2,050 (Bach et al., 2011). Clinically, the syndrome presents with the association of motor slowness (hypokinesia), with muscle rigidity and/or tremor and/or a postural instability (Greenberg et al., 2012). One of the neuropathological hallmarks of PD is a death of dopaminergic neurons in the substantia nigra (SN) *pars compacta*. Neuronal loss begins in the lateral ventral tier of the SN and throughout the illness this remains the most severely affected region. Post-mortem studies have shown that loss of cells from the SN in PD results mainly in profound dopamine (DA) depletion in the motor region of the striatum, nigral projections to the dorsal, and caudal putamen being most affected.

Because a biochemical hallmark of PD is a deficiency of striatal DA, most imaging studies have focused on studying the problem directly, using a variety of methods (Antonini et al., 1997; Constantinescu et al., 2011; Garraux et al., 2013). Measurement of striatal ^18^F-DOPA uptake with PET is still regarded by many as the “gold standard” for the diagnosis of PD. Several studies have reported that patients with sporadic PD have lost up to 50% of normal ^18^F-DOPA uptake from the caudal putamen contralateral to the side with the most severe symptoms compared with 20–30% on the ipsilateral side. The pre-synaptic striatal dopamine deficiency state can be assessed *in vivo* using other nuclear medicine techniques targeted to dopamine transporters (DaT) such as ^123^I-ioflupane (also known by its tradename DaTSCAN) SPECT. These techniques are able to image a pre-synaptic striatal dopamine deficiency state shared by PD and atypical parkinsonian syndromes (APS) such as multiple system atrophy (MSA) (Wenning et al., 2013) and progressive supranuclear palsy (PSP) (Williams and Lees, 2009). They can be used to assist the clinicians to distinguish between these conditions and normal controls, essential and dystonic tremors, drug-induced, and psychogenic parkinsonism. However, it is generally considered that imaging pre-synaptic striatal dopaminergic deficit does not reliably discriminate between PD and APS.

In comparison with PD, the latter are also characterized by a post-synaptic striatal impairment. This can be demonstrated using two main approaches. The first involves ^18^F-FDG PET, which allows assessing the pattern of resting glucose metabolism throughout the brain. Several studies from independent groups consistently showed that FDG uptake pattern in the striatum and other regions can accurately discriminate APS from PD (Ghaemi et al., 2002; Garraux et al., 2013). These studies also showed that ^18^F-FDG PET may also provide some clinically-relevant information for the distinction between APS subgroups. A post-synaptic striatal deficiency state can be specifically demonstrated using radioligands of *D*_2/3_ striatal dopamine receptors. In PD, ^11^C-Raclopride PET studies showed preserved (or even mildly increased in *de novo* cases) striatal binding. In contrast, striatal ^11^C-Raclopride *D*_2_ binding is reduced in APS although significant decreases might only present in around 50% of individuals. Aside from ^11^C-Raclopride, ^123^I-Iodobenzamide (^123^I-IBZM) SPECT has also been used to image post-synaptic striatal dopamine *D*_2/3_ receptors. However, a meta-analysis of IBZM-SPECT studies showed that its negative predictive value was low (Vlaar et al., 2007), while another study suggest that its diagnostic accuracy was considerably lower than that of ^18^F-FDG PET (Hellwig et al., 2012). Finally, studies showed that ^11^C-Raclopride PET and ^18^F-FDG-PET have comparable discrimination accuracy performance for the distinction between PD and MSA (Antonini et al., 1997). One of the drawbacks of ^11^C-Raclopride is its limited clinical availability due to the short physical half-life of ^11^C (20 min), what requires to have a local cyclotron or radiochemistry unit in the neuroimaging center. This impediment to the wider use of PET for clinical dopamine receptor studies could be overcome through the use of suitable ^18^F-labeled radioligands (la Fougère et al., 2010). Thus, ^18^F-desmethoxyfallypride (^18^F-DMFP) has recently emerged as a possible alternative to ^11^C-Raclopride. Its relatively long physical half-life (109 min) enables clinical applications for differentiating between PD and APS. A study found that differences in striatal binding in the posterior putamen between PD and APS provided the larger diagnostic performance using this technique (la Fougère et al., 2010). Furthermore, as compared with IBZM and ^11^C-Raclopride, ^18^F-DMFP enables *D*_2/3_ imaging in extrastriatal regions but this property has not been exploited for the distinction between PD and APS.

Neuroimaging data used to assist the diagnosis of parkinsonism is often analyzed by means of proprietary software to delimit regions of interest (ROIs) and quantify the radiopharmaceutical uptake (Morton et al., 2005; Tossici-Bolt et al., 2006). More sophisticated systems based on machine learning were proposed for FDG PET (Garraux et al., 2013) and DaTSCAN (Prashanth et al., 2014). For the latter modality, several machine learning approaches were presented. For example, in Illán et al. (2012) the voxel intensities of DaTSCAN neuroimages were used as features along with several classifiers to separate PD patients and controls subjects. In Towey et al. (2011) and Segovia et al. (2012) two methods based, respectively, on Principal Component Analysis and Partial Least Squares were proposed to extract relevant features from DaTSCAN data. Structural data were also used to assist the diagnosis of PD (Rana et al., 2015), including the separation of PD and APS (Salvatore et al., 2014). However, the validity of DMFP data to feed statistical classification procedures is still poorly covered. The intensity profile of this imaging modality is meaningfully different of those for DaTSCAN or FDG. In the latter case, the intensity is distributed over a large number of regions, conversely for DaTSCAN most of the signal is gathered in the striatum. DMFP is half-way between DaTSCAN and FDG in terms of its intensity profile: a large proportion of the activity is located in the striatum but other regions also have intensity values large enough to contain patterns allowing the inter-subject discrimination.

In this work, we present a model to automatically distinguish between idiopathic parkinsonism and the non-idiopathic variants using DMFP data. It is based on the application of a machine learning algorithm that consider several regions of interest by means of a multiple kernel approach. We evaluated the proposed model for both, the binary separation of idiopathic and non-idiopathic parkinsonsim and the multigroup separation of PD, MSA, and PSP patients. In addition, we studied the usefulness of combining DMFP and DaTSCAN data in a single computer system. This work is intended to be the baseline of multivariate analyses of DMFP data.

## 2. Materials and methods

### 2.1. Ethics statement

Each patient (or a close relative) gave written informed consent to participate in the study and the protocol was accepted by the Ethics Committee of the University of Munich. All the data were anonymized by the clinicians who acquired them before being considered in this work.

### 2.2. Subjects

Data from 87 subjects showing parkinsonian movement disorders were used for testing purposes (demographic details and groups distribution are gathered in Table 1). Subjects on medication with drugs that have (or are suspected to have) effects on dopaminergic transporters were excluded. A ^18^F-DMFP PET image and a DaTSCAN SPECT image were collected for each subject during the first visit. The former images were acquired 60 min after the ^18^F-DMFP injection. Three (3) frames of 10 min each were recorded using a Siemens/CTI camera. Images were reconstructed as 128 × 128 matrices of 2 × 2 mm voxels by filtered backprojection using a Hann filter with a cutoff frequency of 0.5 Nyquist. The scattered and random events as well as dead time issues were also corrected (Turkington, 2011). After verification of the absence of important head motion between frames (patients had their head immobilized during the emission recording), the three frames were summed and pre-processed (la Fougère et al., 2010). DaTSCAN data were acquired using a GE Healthcare camera and according to widely accepted criteria (Koch et al., 2005).

**Table 1:** **Demographic details of the patients considered in this work (μ and σ stand for the mean and the standard deviation, respectively)**.

	**#**	**Sex**		**Age**		
		**M**	**F**	**μ**	**σ**	**Range**
PD	39	22	17	61.38	11.14	35–81
MSA	24	20	4	68.42	10.73	43–85
PSP	24	12	12	69.29	7.33	55–84

All patients were followed clinically for approximately 2 years after SPECT and PET examinations, at which time the clinical differential diagnoses were assessed by clinicians on the basis of last observations and according to the United Kingdom Parkinson Disease Society Brain Bank Diagnostic Criteria for Parkinson Disease (Hughes et al., 2002) and the second consensus statement on the diagnosis of multiple-system atrophy (Gilman et al., 2008) as well as the established criteria for the diagnosis of progressive supranuclear palsy (Litvan et al., 1996). According to these criteria, 39 patients were labeled as idiopathic parkinsonism and the remaining 48 subjects either MSA or PSP. It is worth noting that all the images were acquired during the first examinations and, therefore, they correspond to early stages of the disorders.

### 2.3. Data pre-processing

After the image reconstruction the images were spatially normalized using the template matching approach implemented in the Statistical Parametric Mapping (SPM) version 8 (Friston et al., 2007). This procedure ensures that any given voxel in different images refers to the same anatomical position across the brains. It was based on the affine part of the SPM normalization procedure, a method that assumes a general affine model with 12 parameters and a Bayesian framework that maximizes the product of the prior function (which is based on the probability of obtaining a particular set of zooms and shears) and the likelihood function (derived from the residual squared difference between the template and the source image) (Ashburner et al., 1997). In order to build the templates for DMFP and DaTSCAN data, only the neuroimages from the idiopathic group was used because of this group is more homogeneous, whereas the non-idiopathic one is the union of the MSA and PSP patients. Thus, idiopathic PD data were first registered to a randomly chosen one. The resulting images and their hemisphere midplane reflections (ensuring a symmetric template) were then averaged and smoothed (8 mm FWHM Gaussian kernel) before being used to spatially normalize the whole set of images. As a result, we got brain volumes with 79 × 95 × 68 voxels of 2 × 2 × 2 mm.

In addition, after the spatial normalization the intensity of the images were also normalized to a value *I*_*max*_, obtained by averaging the 0.1% of the highest intensities per image, as described in Saxena et al. (1998).

### 2.4. Multivariate analysis based on machine learning

Unlike univariate analyses where each voxel is independently analyzed, multivariate approaches analyze a neuroimage as a whole and explicitly consider the relationships between voxels (Schrouff et al., 2013). Effects comprised in the data, including activations and confounding and errors effects are assessed statistically both at each voxel and as interactions between voxels (Friston and Büchel, 2007).

A large proportion of multivariate analyses for neuroimaging data are based on statistical classification, such as Support Vector Machine (SVM) (Vapnik, 1998). During the training step, a binary statistical classifier builds a function *f*: ℝ^*N*^ → ±1 using a set of labeled samples so that *f* is able to predict the label of new unseen samples. SVM calculates this function by estimating the hyperplane that has the largest distance to the closest sample with any label. Then, the label of a new sample is estimated according to the side of the hyperplane in which the new sample is. Mathematically, the decision hyperplane (also named maximal margin hyperplane) is defined as:

(1) $$g(\text{x})={\text{w}}^{T}\text{x}+w_{0}=0,$$

where **w** is the weight vector, orthogonal to the decision hyperplane, and *w*_0_ is the bias term. Calculating the hyperplane involves solving the following problem:

(2) $$\begin{array}{l} \text{minimize }\frac{1}{2}∥\text{w}{∥}^{2}+\;C\sum_{i\;=\;1}^{N}\xi_{i}\\ \text{subject to }y_{i}({\text{w}}^{T}\text{x}+w_{0})\geq 1-\xi_{i},\;i=1,2,\ldots ,N \end{array}$$

where *C*, *N*, and ξ_*i*_ stand for a predefined trade-off parameter between model simplicity and classification error, the number of training instances and the slack variables, respectively. This problem can be simplified by applying Lagrangian functions and results in:

(3) $$\begin{array}{l} \text{maximize}\sum_{i=1}^{N}\alpha_{i}-\frac{1}{2}\sum_{i=1}^{N}\sum_{j=1}^{N}\alpha_{i}\alpha_{j}y_{i}y_{j}k({\text{x}}_{i},{\text{x}}_{j})\\ \text{subject to}\sum_{i=1}^{N}\alpha_{i}y_{i}=0\\ \;0\leq \alpha_{i}\leq C,\;i=1,2,\ldots ,N \end{array}$$

where α is the vector of dual variables corresponding to each separation constraint, and *k*(**x**_*i*_, **x**_*j*_) is a function ℝ^*D*^ × ℝ^*D*^ → ℝ known as “kernel” (Müller et al., 2001). For linear SVM, *k*(**x**_*i*_, **x**_*j*_) = **x**_*i*_**x**_*j*_.

### 2.5. Analysis based on multiple kernel learning

After the pre-processing steps, the data were automatically parceled using the well-known Automated Anatomical Labeling (AAL) atlas (Tzourio-Mazoyer et al., 2002). This procedure allows isolating specific regions such as the striatum or the olfactory bulb, and that way, they can be independently analyzed.

**Figure 1:**
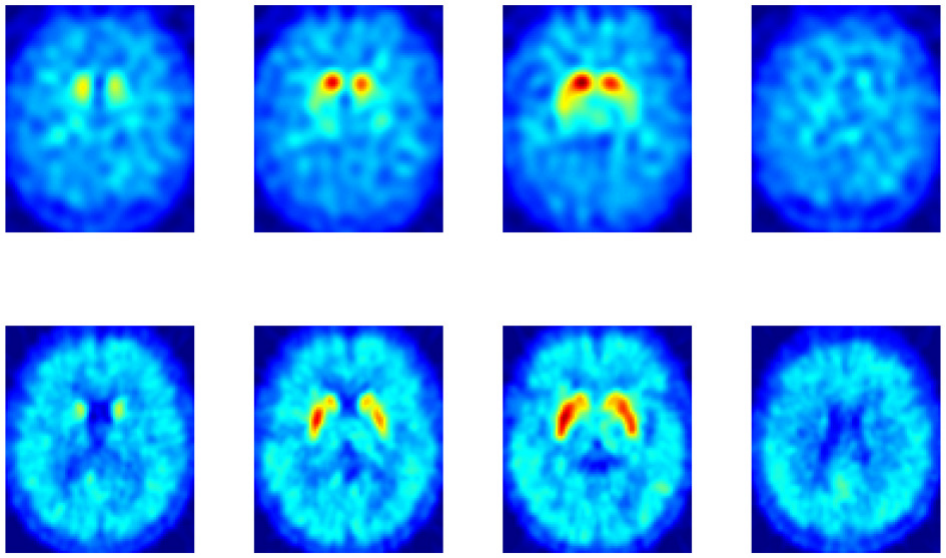
**Comparison between a DaTSCAN (top row)** and a DMFP **(bottom row)** neuroimage from a patient diagnosed with Parkinson's disease.

Neuroimaging from both DaTSCAN and DMFP radioligands gathers most of the total activation in the striatum. However, DMFP-based data also contain information in other regions (see Figure 1). There is therefore a reasonable prospect that DMFP data contain useful information for a diagnosis in areas outside the striatum. This would be overlooked during the visual examination of the images, while automatic procedure (based on machine learning) may efficiently exploit this information. In order to better account for the different scales and relevances of the various regions, we used a multiple kernel learning (MKL) procedure along with a SVM classifier.

In a MKL procedure, two or more kernels are built from feature subsets and then combined by means of a predefined function (Gönen and Alpaydın, 2011). In this work, we used a kernel per each region of interest and a linear combination function that provides a good performance with relatively low computational burden:

(4) $$k({\text{x}}_{i},{\text{x}}_{j})=\sum_{m=1}^{N_{k}}q_{m}k_{m}({\text{x}}_{i}^{m},{\text{x}}_{j}^{m})$$

where *N*_*k*_ is the number of kernels; *q*_*m*_ stands for the weight of kernel *k*_*m*_ (estimated through cross-validation); **x**_*i*_, **x**_*j*_ are two feature vectors and ${\text{x}}_{i}^{m}$, ${\text{x}}_{j}^{m}$ are subset of **x**_*i*_, **x**_*j*_ with only the features used for kernel *k*_*m*_.

### 2.6. Identifying regions of interest

A linear SVM trained classifier allows us to analyze the weight assigned by the classifier to each feature included in the training set (related with importance of that feature in the separation problem). This information can be directly extracted from the weight vector, **w**, defined in Equation (1). Thus, using all the brain voxels as feature we estimated the importance of each voxel in the separation problem. Then, the weight/importance of each region was computed as

(5) $$w_{r}=\frac{\sum_{i\;=\;1}^{N_{r}}w_{x_{i}}}{N_{r}}\;\forall x_{i}\in r$$

where *w*_*x*_*i*__ is weight corresponding to the voxel *x*_*i*_ and *x*_*i*_, *i* = 1, …, *N*_*r*_ are the voxels in the region *r*.

**Figure 2:**
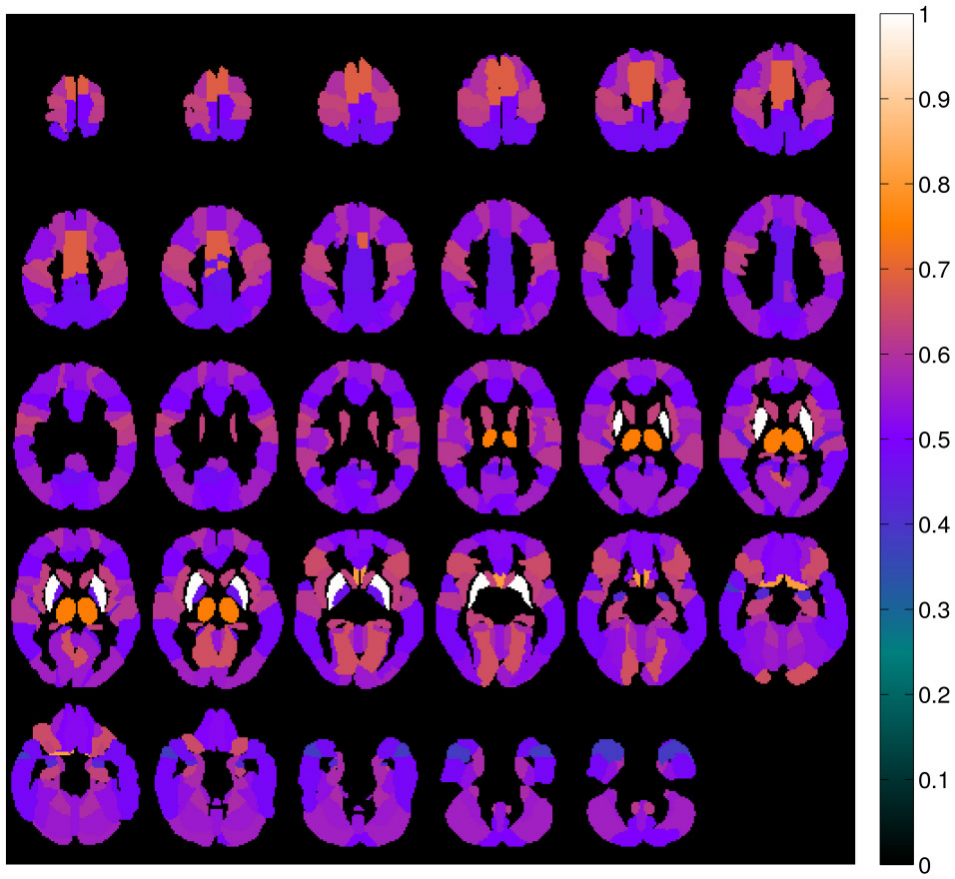
**Importance of each brain region for the classification problem**. The values were estimated from the voxels weight computed by a linear SVM classifier, and normalized to the range [0, 1].

**Figure 3:**
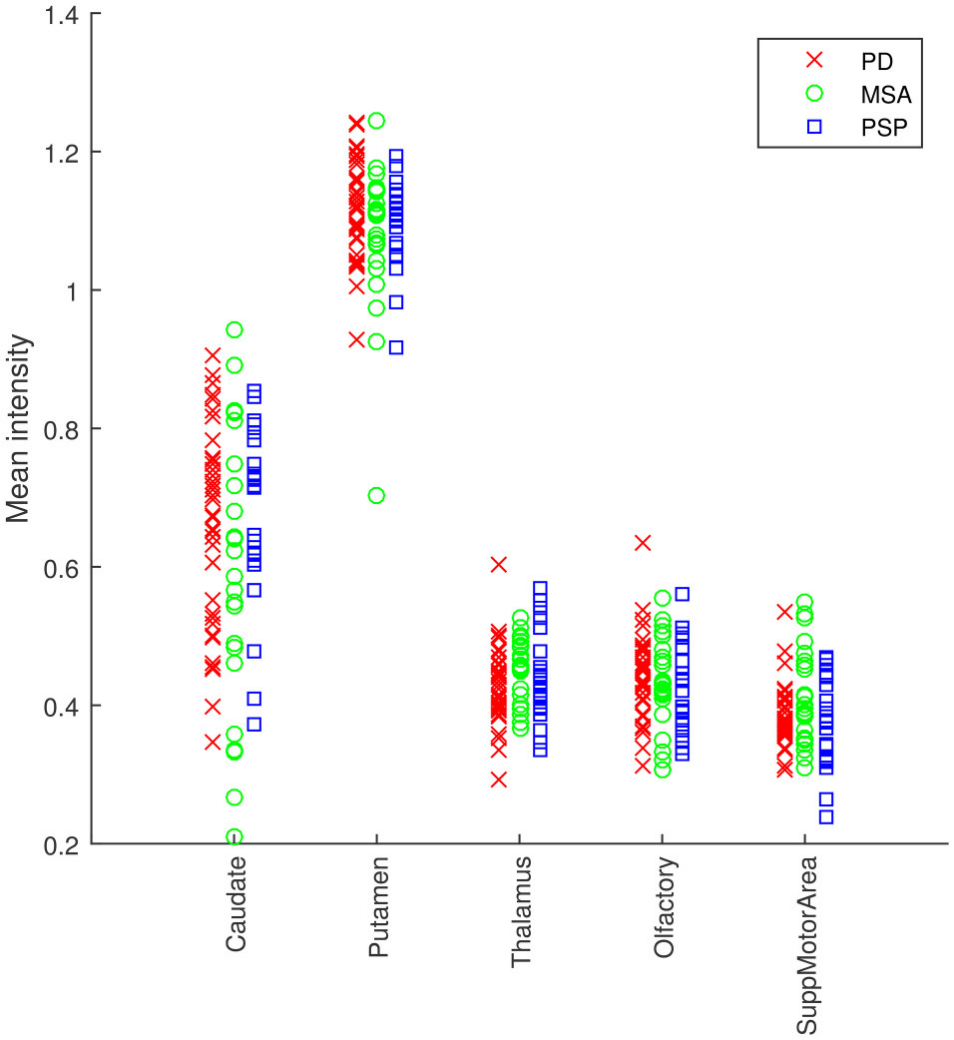
**Analysis of the average intensity of the five regions of interest found in the analysis of DMFP data**. Each patient is represented by five values: the average intensity of his DMFP neuroimage on the caudate, putamen, thalamus, olfactory, and supplementary motor area. The values of each region are grouped by the patient group: PD (red crosses), MSA (green circles), and PSP (blue squares).

A map containing the voxel weights (rearranged into brain form) is shown in Figure 2. It was computed by training a binary SVM classifier (linear kernel) with DMFP data from PD patients in one group and data from MSA and PSP patients in other group. As expected, the most important region is the striatum, specifically the putamen area. The high weight assigned to other regions such as the olfactory bulb or the thalamus and the supplementary motor area is also interesting. Figure 3 shows the mean intensity of these five areas for all the DMFP neuroimages in our dataset. Note that the mean intensity of the putamen is largely higher than those of the thalamus, olfactory bulb, and supplementary motor area. These differences were successfully addressed by the MKL-based proposed approach. Figure 3 also suggests that there is no important differences between groups when only the mean of the pre-selected ROIs is analyzed.

## 3. Experiment and results

Several experiments were conducted in order to evaluate the application of the multivariate approach described above to our DMFP dataset. Initially, we address the binary diagnosis problem, i.e., the separation of two groups: idiopathic and non-idiopathic (includes MSA and PSP disorders) parkinsonism. Table 2 shows the classification measures obtained in this case and compares the results obtained by the proposed MKL approach with the ones achieved by classical approaches such as using only the voxels at the striatum or using all the voxels inside the brain. The accuracy, sensitivity and specificity of the systems were estimated using a *k*-fold (*k* = 10) cross-validation scheme. All the systems were based on SVM classification and the parameters (trade-off parameter *C* and kernel weights *q*_*m*_ for MKL approaches) were estimated using a grid search (Varma and Simon, 2006). Values *C* = 2^*e*^, *e* = {−3, −2, …, 5} and *q*_*m*_ = {0.1, 0.2, …0.9} satisfying $\overset{N_{k}}{\underset{m=1}{\sum }}q_{m}=1$ were assessed.

**Table 2:** **Accuracy, sensitivity, and specificity obtained by the proposed approach and other classical approaches when separating neuroimaging data from idiopathic and non-idiopathic parkinsonism**.

	**Accuracy**	**Sensitivity**	**Specificity**
Using DMFP data:			
Voxels in the striatum (%)	68.96	79.17	56.41
All the voxels inside the brain (%)	67.82	75.00	58.97
MKL approach (five regions) (%)	73.56	77.08	69.23
Using DaTSCAN data:			
Striatum voxels (%)	59.77	62.50	56.41
Using DMFP and DaTSCAN data:			
Voxels in the striatum (%)	70.11	77.08	61.54
All the voxels inside the brain (%)	63.22	70.83	53.85
MKL approach (six regions) (%)	72.41	75.00	69.23

In order to avoid biased results, the selection of the regions used in the MKL approach was performed using only the training dataset, i.e., in each iteration of the cross-validation loop, we selected the five regions with the most weight (note that the striatum is divided in two regions in the AAL atlas: putamen and caudate nucleus). The putamen, caudate nucleus, olfactory bulb, and thalamus were among the five selected regions in all the iterations. The supplementary motor area was the fifth region in 92% of all cases. The pseudo-code corresponding to this experiment is shown in Algorithm 1.

**Algorithm 1:**
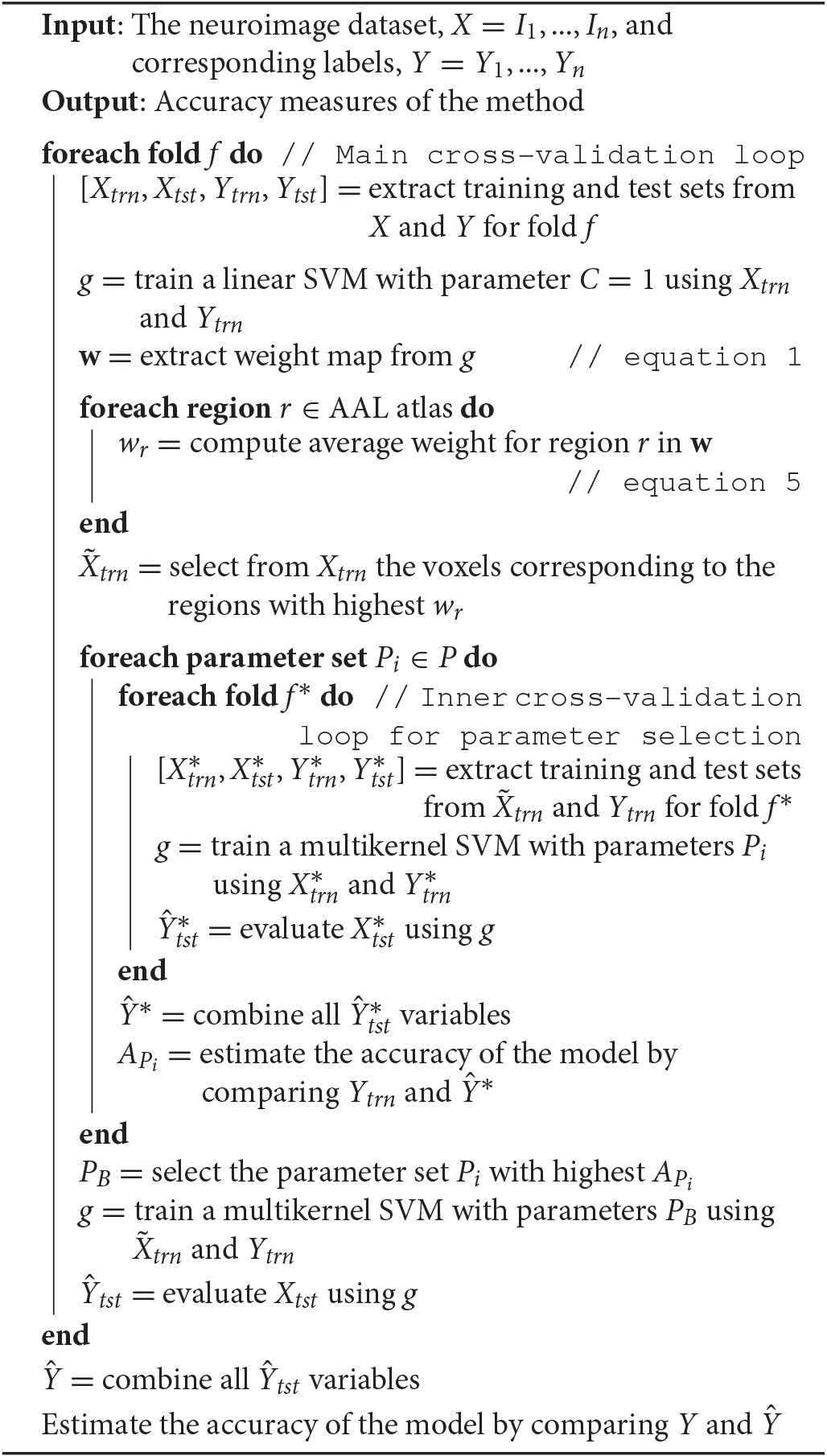
**Evaluation procedure**.

*P_i_* = {C, *q*_1_, *q*_2_, …, *q_N_k__*} denotes the parameter set required by the multikernel SVM classifier. *C* stands for the trade-off parameter of the SVM algorithm (Equation 2) whereas *q_m_, m* = 1, 2, …, *N_k_* is the weight of the kernel *k_m_* (Equation 4). *P* gathers all *P_i_* sets, i.e. all possible combinations of kernel weights and values for parameter *C*.

**Figure 4:**
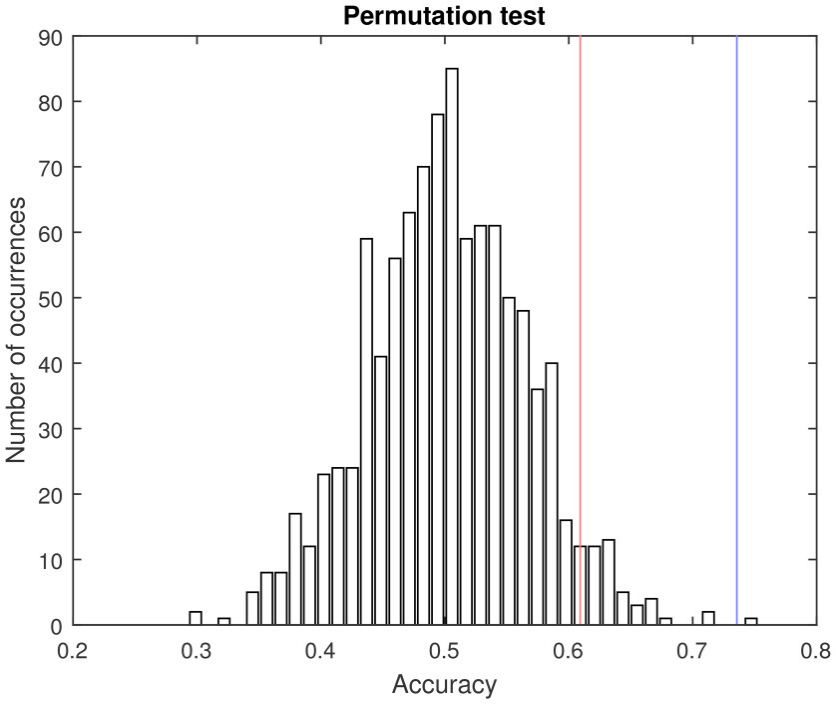
**Pemutation test**. Histogram of the accuracy rates achieved by using randomly generated label sets (1,000 repetitions) and the proposed multikernel-based method. Red and blue lines are, respectively, the accuracy associated with a *p*-value of 0.05 and the accuracy obtained when using the true labels (73.56%).

As shown in Table 2, the proposed method based on multiple kernel learning achieved an accuracy rate of 73.56%. The statistical significance of this measure was assessed by means of a permutation test. In this procedure, the classification algorithm was run 1,000 times using different label sets (generated as random permutations of the original label set). A *p*-value was then calculated as the fraction of the executions in which the accuracy was greater than or equal to the accuracy observed when using the correct labels (Pereira et al., 2009). As a result, a *p*-value of 0.001 was obtained. Figure 4 shows the histogram corresponding to the accuracy rates obtained in the procedure.

Subsequently, the tree-groups separation problem was addressed. In this case, the system was trained to differentiate between the disorders considered in our study: PD, MSA, and PSP. To this end, an ensemble of SVM classifiers and the one-against-one strategy was used. The results are gathered in Table 3.

**Table 3:** **Accuracy achieved by a multiclass classification system when separating PD, MSA, and PSP neuroimages**.

	**Global acc**.	**PD acc**.	**MSA acc**.	**PSP acc**.
Using DMFP data:				
Voxels in the striatum (%)	56.32	56.41	66.67	45.83
All the voxels inside the brain (%)	49.43	64.10	37.50	37.50
MKL approach (five regions) (%)	66.67	82.05	54.17	54.17
Using DaTSCAN data:				
Striatum voxels (%)	44.83	61.54	29.17	33.33
Using DMFP and DaTSCAN data:				
MKL approach (six regions) (%)	62.07	76.92	54.17	45.83

In both classification approaches, binary and multiclass, the results obtained by using DMFP data were compared with the ones obtained by DaTSCAN neuroimages. For the latter neuroimage modality only the voxels at the striatum were considered as it is common in the field. Additionally, an experiment that combined both neuroimages modalities (DaTSCAN and DMFP) was performed. It allows us to evaluate if the information contained in DaTSCAN neuroimages can complement that contained in DMFP data. To this end, we extend the MKL approach to include six regions: the five regions selected for DMFP data and striatum from the DaTSCAN neuroimages. The results are included in Tables 2, 3.

### 3.1. Univariate analysis

**Figure 5:**
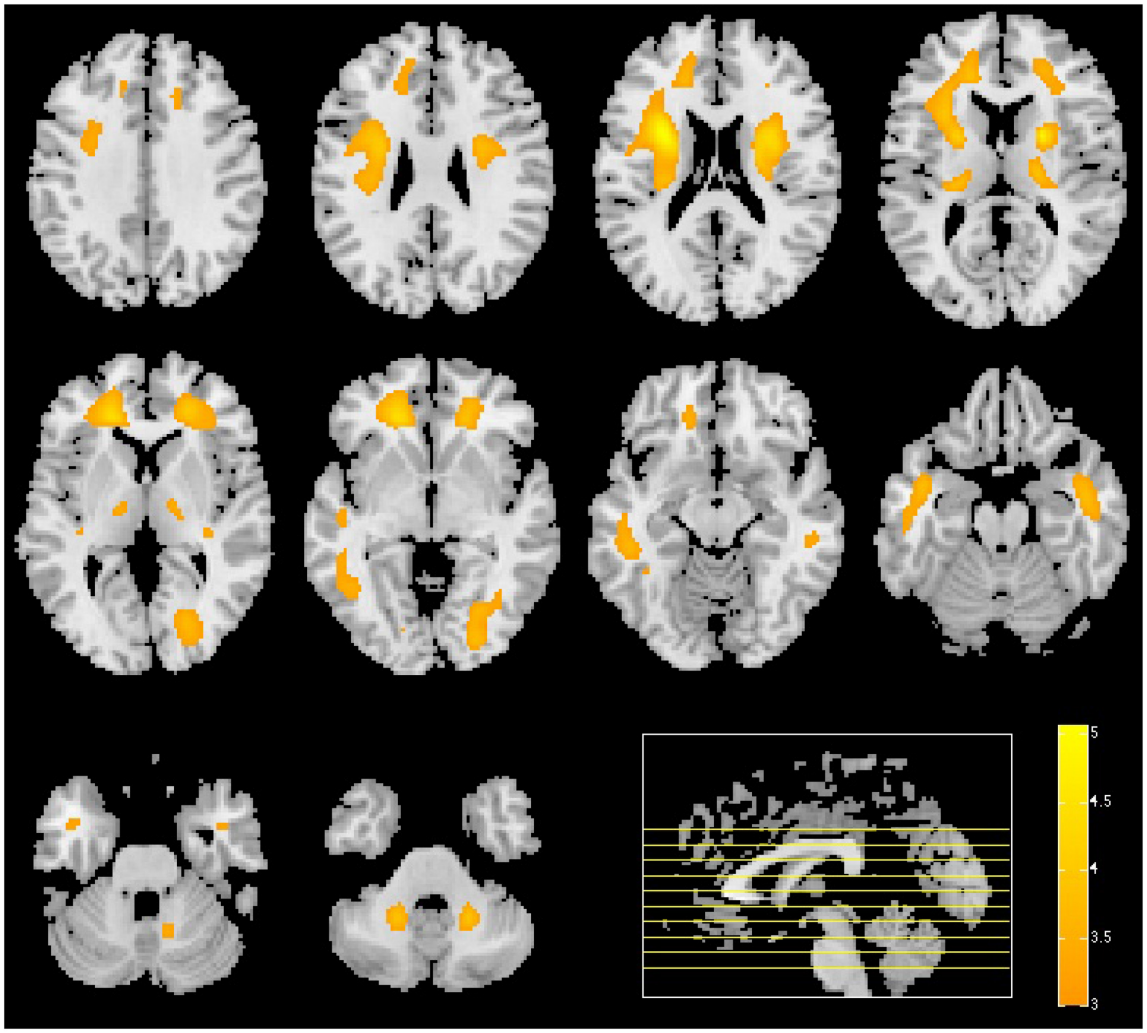
**Result of the univariate analysis**. *t*-test comparing patients with idiopathic and non-idiopathic parkinsonism. Regions in orange/yellow are significantly lower (*p* < 0.001, uncorrected) in idiopathic compared with non-idiopathic patients.

For the sake of completeness, we compared the regions identified in the previous section with the ones obtained by means of a *t*-test. A smoothed version of the DMFP PET neuroimages (Gaussian filter of 8 mm FWHM) was analyzed using a 2-sample *t*-test in SPM. The results indicate that patients with idiopathic parkinsonism have lower dopamine levels than non-idiopathic patients in the striatum, thalamus and supplementary motor area. Specifically, clusters showing significant difference appeared in the thalamus, anterior cingulate cortex and putamen with *z*-values up to 4.74 (see Figure 5). Some differences (*p* < 0.001) also appeared in the supplementary motor area (454 mm^3^) and caudate nucleus (22 mm^3^).

### 3.2. ROC analysis

**Figure 6:**
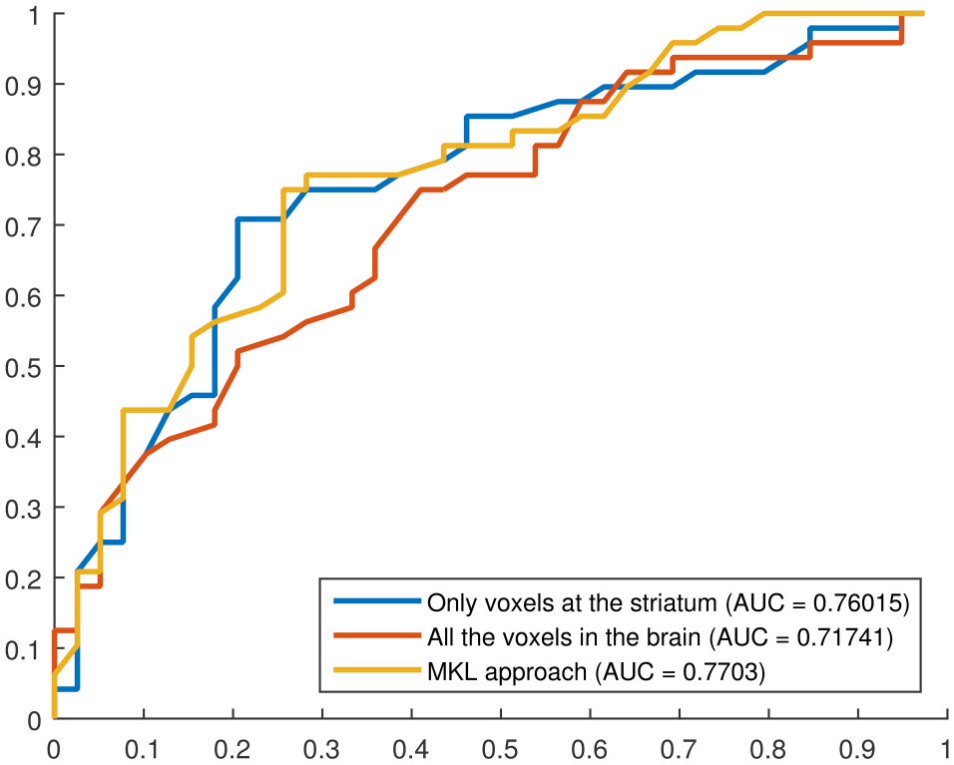
**ROC curves for three binary classification systems using DMFP data: (i) using only the voxels at the striatum, (ii) using all the voxels in the brain, (iii) using the proposed MKL-based approach**. The AUC for each curve is shown in the legend.

Finally, receiver operating characteristic (ROC) curves were computed to assess the trade off between sensitivity and specificity provided by the developed systems. These curves provide an estimation of the performance of a classification procedure, not only in terms of sensitivity and specificity but also in terms of the global performance by measuring the area under the curve (AUC). Curves and AUCs are shown in Figure 6.

## 4. Discussion and conclusions

Separating idiopathic and non-idiopathic parkinsonian patients in an early stage is a challenge because both groups can show similar clinical signs and symptoms (Litvan, 1999). In addition, neuroimaging techniques such as DaTSCAN, widely used to assist the diagnosis of PD, may have difficulty to differentiate between PD and MSA or PSP, as shown in our experiments. In this work, we evaluated the use of ^18^F-DMFP PET in computer systems to distinguish between three parkinsonian syndromes (PD, MSA, and PSP), yielding accuracy rates about 70%. Using a multivariate analysis, we found that the striatum, olfactory bulb, thalamus, and supplementary motor area are the most important regions to separate the groups. These findings were partly corroborated through univariate analysis and are consistent with previous results reported in recent studies. For example, in Chen et al. (2014) the authors found that the volumes of the olfactory bulb and tract were significantly reduced in idiopathic PD patients compared to normal subjects and MSA patients. This corroborate the idea that the mechanisms that produce idiopathic PD also affect this region.

This result obtained by hypothesis free analysis of DMFP data is in line with clinical and histopathological observations. Clinically, olfactory testing can be used to identify patients in the premotor phase of Parkinson's disease (Berardelli et al., 2013). The olfactory deficits is caused by PD pathology in the olfactory bulb and can already be observed stage I of the Braak's staging of PD pathology (Braak et al., 2002). The key role of striatal pathology in MSA and PSP is widely established and also reflected in our hypothesis free approach (Litvan et al., 1997). On the other hand, evidences of the involvement of the motor cortex in PD were reported in Vacherot et al. (2010); Lindenbach and Bishop (2013), although results have been controversial (Viaro et al., 2011). Finally, the relationship between PD and thalamus is not yet proven on pathological bases and therefore might reflect functional disturbances on basal ganglia circuits that involve the thalamus. In short, our experiments corroborate that there exist differences in those regions (olfactory bulb, supplementary motor area, and thalamus) between PD, MSA, and PSP patients.

In this manuscript, we propose a methodology to analyze DMFP data for diagnostic purposes. The primary idea is to use not only the striatum region but additionally to take into account the other regions of the brain. In order to address the small sample size problem and overcome the differences in terms of size between different regions, we propose to use a MKL approach, which is considered to be a good solution to combine heterogeneous data sources into one classification procedure (Gönen and Alpaydın, 2011; Segovia et al., 2014). In some sense, a MKL classification procedure as the one used in this work can be seen as a kind of regularization process that assigns the same weigh to all the voxels in a specific region instead of a different weigh per each voxel as a standard SVM classifier does.

Two classification approaches were addressed. The first one consisted on separating idiopathic and non-idiopathic parkinsonism (binary classification) whereas the second one was able to differentiate between PD, MSA, and PSP data (multiclass classification). The proposed methodology based on MKL classification yields higher accuracy rates than classical approaches in both cases, however these results are limited by the relative small number of patients included in this study. Unfortunately the availability of these kind of data, especially the ones acquired using new or uncommon radiopharmaceuticals is limited. The significance of the accuracy rate achieved by the proposed method was estimated by means of permutation tests obtaining a *p* = 0.001. Recently, Noirhome et al. demonstrated that permutation tests are more adequate than classical approaches as binomial assessment to calculate the significance of classification measures estimated using cross-validation schemes (Noirhomme et al., 2014). It is worth noting the relatively low accuracy rates obtained for MSA and PSP classes in the multiclass approach. These results suggest that the differences between these disorders do not appear in DMFP data. This is not unique to this data modality. Previous studies based on FDG data (Garraux et al., 2013) also faced the same issue. Finally, we included an experiment combining DMFP and DaTSCAN data in the same system. The results showed that including DaTSCAN data helps in the separation problem when only the striatum voxels were used however, when the additional DMFP regions proposed in this work (the putamen, caudate nucleus, olfactory bulb, thalamus, and supplementary motor area) were included (in the MKL-based approach) DaTSCAN data did not increase the separation ability of the classifier. All in all, DaTSCAN data is probably not necessary to differentiate between PD, MSA, and PSP patients if we use new systems based on DMFP data that take advantage of all the information contained in these neuroimages (not only the striatum).

## Author contributions

Conception or design of the work: FS, JG, JR, FM, JL, GG, CP. Data collection: JL, MS, MB, AR, KB. Data analysis and interpretation: FS, JG, JR, FM, CP. Drafting the article: FS, JG, JR, FM, GG, CP. Critical revision of the article: FS, JG, JR, JL, CP.

### Conflict of interest statement

The authors declare that the research was conducted in the absence of any commercial or financial relationships that could be construed as a potential conflict of interest.
